# The effects of exercise training for eight weeks on immune cell characteristics among breast cancer survivors

**DOI:** 10.3389/fspor.2023.1163182

**Published:** 2023-05-11

**Authors:** Ainhoa Arana Echarri, Lauren Struszczak, Mark Beresford, John P. Campbell, Dylan Thompson, James E. Turner

**Affiliations:** ^1^ Department for Health, University of Bath, Bath, United Kingdom; ^2^ Department for Oncology and Haematology, Royal United Hospitals Bath NHS Trust, Bath, United Kingdom; ^3^ School of Sport, Exercise and Rehabilitation Sciences, College of Life and Environmental Sciences, University of Birmingham, Birmingham, United Kingdom

**Keywords:** breast cancer, survivorship, immune profiles, anti-cancer immunity, lifestyle, exercise, body composition

## Abstract

**Methods:**

This study examined the effects of exercise training for 8 weeks on blood immune cell characteristics among 20 breast cancer survivors (age 56 ± 6 years, Body Mass Index 25.4 ± 3.0 kg m^2^) within two years of treatment. Participants were randomly allocated to a partly-supervised or a remotely-supported exercise group (*n* = 10 each). The partly supervised group undertook 2 supervised (laboratory-based treadmill walking and cycling) and 1 unsupervised session per week (outdoor walking) progressing from 35 to 50 min and 55% to 70% V˙O_2_max. The remotely-supported group received weekly exercise/outdoor walking targets (progressing from 105 to 150 min per week 55% to 70% V˙O_2_max) via weekly telephone calls discussing data from a fitness tracker. Immune cell counts were assessed using flow cytometry: CD4+ and CD8+ T cells (Naïve, NA; Central memory, CM; and Effector cells, EM and EMRA; using CD27/CD45RA), Stem cell-like memory T cells (TSCMs; using CD95/CD127), B cells (plasmablasts, memory, immature and naïve cells using CD19/CD27/CD38/CD10) and Natural Killer cells (effector and regulatory cells, using CD56/CD16). T cell function was assessed by unstimulated HLA-DR expression or interferon gamma (IFN-γ) production with Enzyme-linked ImmunoSpot assays following stimulation with virus or tumour-associated antigens.

**Results:**

Total leukocyte counts, lymphocytes, monocytes and neutrophils did not change with training (*p* > 0.425). Most CD4+ and CD8+ T cell subtypes, including TSCMs, and B cell and NK cell subtypes did not change (*p* > 0.127). However, across groups combined, the CD4+ EMRA T cell count was lower after training (cells/µl: 18 ± 33 vs. 12 ± 22, *p* = 0.028) and these cells were less activated on a per cell basis (HLA-DR median fluorescence intensity: 463 ± 138 vs. 420 ± 77, *p* = 0.018). Furthermore, the partly-supervised group showed a significant decrease in the CD4+/CD8+ ratio (3.90 ± 2.98 vs. 2.54 ± 1.29, *p* = 0.006) and a significant increase of regulatory NK cells (cells/µl: 16 ± 8 vs. 21 ± 10, *p* = 0.011). T cell IFN-γ production did not change with exercise training (*p* > 0.515).

**Discussion:**

In summary, most immune cell characteristics are relatively stable with 8 weeks of exercise training among breast cancer survivors. The lower counts and activation of CD4+ EMRA T cells, might reflect an anti-immunosenescence effect of exercise.

## 1. Introduction

Breast cancer is the most commonly diagnosed cancer globally (1, 2). Survival over 5-years is close to 90% in most western nations (3–5) due to advances in screening, early detection and treatment (6). As breast cancer is the most prevalent cancer in many countries and the number of people living with and beyond breast cancer is increasing (7), there is a growing need for interventions—such as exercise and physical activity—that reduce the risk of recurrence, mortality and other comorbidities developing. Indeed, this need is recognised globally, as shown by the publication of cancer-specific exercise and physical activity guidelines or position stands by the World Health Organisation (WHO), the American Cancer Society (ACS), the American Society of Clinical Oncology (ASCO), the Clinical Oncology Society of Australia (COSA), the World Cancer Research Fund International (WCRF) and the American College of Sports Medicine (ACSM) (8–13).

There is now good evidence that exercise and physical activity undertaken before or after a cancer diagnosis improves overall health, reduces risk of recurrence, and increases survival (14–18). For example, a study of 959 breast cancer patients showed that 2.5 h or more of brisk walking each week was associated with a 44% lower mortality risk compared with no physical activity (19). Furthermore, a study of 1,340 breast cancer patients showed that those who met physical activity guidelines (150 min of moderate-intensity physical activity each week) both before diagnosis and after treatment had a 55% lower risk of recurrence and a 68% lower risk of all-cause mortality (20). Indeed, the latest WCRF review and meta-analysis as part of the continuous update project, concluded that the most active patients with breast cancer have 42% lower cancer-specific mortality risk than the least active patients (21). However, in most guidelines, the dose of exercise or physical activity, was originally defined in cancer prevention studies, and more research is needed among people living with and beyond cancer (22). In particular, the mechanisms by which exercise or physical activity reduce cancer recurrence and mortality are not understood, although processes that involve changes to inflammation and immunity are possible candidates (18, 23–25).

Chronic inflammation overall and the action of specific inflammatory processes and cytokines can facilitate tumour progression (26). Moreover, inflammation exacerbates ageing, referred to as inflammageing, contributing towards immunosenescence (27). Given that these processes are influenced by exercise and physical activity, countering inflammation and limiting immunosenescence are possible lifestyle-related anti-cancer mechanisms (28–30). In support, regular exercise and physical activity over months and years can modulate immune profiles, for example, by limiting the accumulation of cell populations linked with immunosenescence (31) and reducing inflammation (32). In addition, individual exercise bouts mobilise leukocytes, and sub-populations, enhancing immunosurveillance (33). Many exercise-mobilised cells are differentiated T cells and NK cells that are cytotoxic against tumour cells (34, 35). Given that most breast tumours are immunogenic (36, 37), T cells represent a likely mechanism through which exercise exerts anti-cancer effects (30, 38).

Most studies examining exercise-induced anti-cancer mechanisms have been pre-clinical mouse models. Studies recruiting people living with and beyond cancer that incorporate measurements relevant to cancer immune-surveillance are less common, but some understanding comes from studies among human populations considered to be at high risk of cancer. For example, one study recruited 33 postmenopausal women considered to be at high risk of breast cancer (39). Participants were randomized to either 12 weeks of high intensity interval training (HIIT), moderate-intensity continuous training (MICT) or usual care (UC) and a primary outcome was NK cell cytotoxicity against tumour cell lines. There were no statistically significant differences in NK cell cytotoxicity with exercise training irrespective of group (39). However, across all participants, low baseline cardiorespiratory fitness was negatively associated with exercise-induced increases in NK cell cytotoxicity, implying that less fit individuals might benefit most from exercise (39). In a sub-sample of 24 participants, T cell profiles were also examined (40). It was concluded that exercise alters the frequency of cells associated with immunosenescence, but each exercise mode elicited divergent effects. HIIT was interpreted as invoking pro-immunosenescent effects, by decreasing CD4+ T cells, CD4+ Naïve T cells, CD4+ recent thymic emigrants and the CD4+/CD8+ T cell ratio. However, MICT was interpreted as invoking anti-immunosenescent effects, by increasing total lymphocytes and CD8+ effector memory T cells, and there was a greater positive change in total T cells, CD4+ Naïve T cells, CD4+ central memory T cells, and CD4+ recent thymic emigrants in the MICT group (40).

Further research is needed to understand potential anti-immunosenescence and anti-cancer effects of exercise and physical activity among people living with or beyond cancer, incorporating functional measurements previously shown to correlate with clinical outcomes (41). Therefore, the present study examined the effect of exercise training over eight weeks on the phenotype of circulating T cells, B cells and NK cell populations among breast cancer survivors within 2 years of treatment. Importantly, this study also made functional measurements, including basal unstimulated T cell activation, and T cell recognition of virus and tumour-associated antigens.

## 2. Methods

### 2.1. Participants

This study comprised 20 participants. Inclusion criteria were; women aged between 35 and 69 years, body mass index 20–35 kg m^2^, post-menopausal (or who had not had a menstrual period for at least 1 year), and a past diagnosis of non-metastatic non-bilateral stage I–III invasive breast cancer. Participants had received their last treatment at least 2 months before enrolment, but no longer than 5 years prior, however women on long-term endocrine therapy were eligible. Participants were free from cancer, significant cardiac comorbidity, severe hypertension (>200/120 mmHg) or cardiovascular disease and did not have an active infection at the time of enrolment. Participants self-reported to not undertake regular structured physical activity for more than 30 min on two or more occasions per week. Participants were recruited via an NHS hospital in the UK and provided full and informed consent. The study was approved by an NHS research ethics committee (18/WA/0314).

Participants were 56 ± 6 years of age, with body mass 67 ± 8 kg, Body Mass Index 25.4 ± 3.0 kg m^2^ and V˙O_2_max 29.07 ± 5.55 ml kg^−1^ min^−1^. Table 1 and Supplementary Table S1 show the diagnostic and treatment history of participants. Upon recruitment, time since diagnosis was 14 ± 7 months and time since surgery was 12 ± 6 months. All participants had received a diagnosis of primary invasive breast cancer, with 30% of participants diagnosed with ductal carcinoma *in situ* and 10% of participants with multifocal carcinoma. 65% of participants had tumours which were positive for Estrogen/Progesterone Receptors (ER+/PR+) and 10% were positive for Human Epidermal Growth Factor Receptor 2 (HER2). None of the patients had advanced metastatic disease. All participants had surgery, and some underwent radiotherapy (75%), hormone therapy (60%), chemotherapy (25%) or immunotherapy (10%).

**Table 1:** Diagnostic and treatment information.

Clinical summary	% of participants (number or sub-type) or duration
Diagnosis	
Ductal carcinoma *in situ* (DCIS)	30% (6/20)
Multifocal carcinoma	10% (2/20)
Hormone expression (ER+/PR+)	65% (13/20)
HER2 expression (HER2+)	10% (2/20)
Tumour Grade (G)	0% (G1), 45% (G2), 25% (G3)
TNM scoring	
Tumour (T)	35% (T1), 30% (T2), 5% (T3)
Nodes (N)	75% (N0), 15% (N1), 5% (N2), 5% (N3)
Metastasis (M)	100% (M0)
Average time since diagnosis	14 ± 7 months
Treatment	
Surgery	100% (20/20)
Chemotherapy	25% (5/20)
Radiotherapy	75% (15/20)
Hormone therapy	60% (12/20)
Anti- HER2+ therapy	10% (2/20)
Average time since surgery	12 ± 6 months

Data is expressed as percentage of participants or as mean ± SD. Grade refers to the histologic grade (G1, G2, G3, etc). TNM staging defines the characteristics of the tumour 0 to 4 (T) and 0 to 3 (N). ER, Estrogen receptor; HER2, Human Epidermal growth factor Receptor 2, PR, progesterone receptor.

### 2.2. Study design

Participants were randomised to one of two intervention groups (allocation ratio 1:1, block size 6) by an independent researcher using a computer-generated randomisation list. Randomisation was stratified for previous chemotherapy treatment (yes/no) and BMI (<25 kg m^2^ or >25 kg m^2^). The two intervention groups were either an eight-week partly-supervised exercise group, or an eight-week remotely-supported exercise group (see Section 2.5).

### 2.3. Participant characterisation

Measurements were made before the intervention (within 7 days) and at least 24 h after the last exercise bout (within 7 days). Participants visited a research laboratory in the morning (between 07:00 and 11:00) after fasting overnight (since 22:00) and having refrained from alcohol, caffeine or exercise in the previous 24 h. After a 10 min seated rest, blood pressure was measured with an automated sphygmomanometer (Diagnostec EW3106, Panasonic, Japan) in the contralateral arm to the affected breast. Height was assessed with a wall mounted stadiometer (Holtain Ltd, UK), body mass was assessed using electronic scales (BC-543 Monitor, Tanita, Tokyo, Japan) and waist and hip circumference was measured using a tape (SECA 201, Hamburg, Germany). Body composition was assessed using a dual energy x-ray absorptiometry (DEXA) whole body scan (QDR, Discovery W, Hologic, Bedford, UK).

Cardiorespiratory fitness was assessed using a treadmill-based maximal walking test to exhaustion (HP Cosmos Saturn, Nußdorf, Germany) comprising 3 min stages, beginning at 2.7 kph with a 1% gradient, and increasing by 1.3 kph until 6.6 kph, with further intensity increments via increasing gradient by 2%. During the final minute of each stage, heart rate was measured using telemetry (Polar heart rate monitor RS400, Kempele, Finland), rating of perceived exertion (RPE) was recorded using the Borg scale, and an expired air sample was collected using Douglas bags. Oxygen and Carbon dioxide concentrations were assessed with a Servomex 5200 Multi-Purpose HF gas analyser (Servomex; Sussex, UK). The volume of air was measured with a dry gas meter (Harvard Apparatus, Cambridge, UK).

Physical function was assessed using three tests. First, for the 6 min walk test, participants walked as far as possible in 6 min between two cones placed 7 meters apart (42). Second, for the sit-to-stand test, participants performed as many sit-to-stands in 30 s (seated on a standard chair, rising to reach full knee extension, and return to seated, with arms folded across the chest) (43, 44). Third, for the 8-foot get-up-and-go test, participants rose from a seated position on a standard chair, walked 8-feet, returning to a seated position as quickly as possible (45, 46).

### 2.4. Blood sample collection, processing and storage

After a 10 min seated rest, approximately 30 ml of blood was collected into a syringe containing sodium heparin (4 IU/ml) for isolation of peripheral blood mononuclear cells (PBMCs). A further 5 ml of blood was collected into an anticoagulant-free serum separation tube and left to clot at room temperature for 30 min, and 5 ml of blood was collected into a potassium–ethylenediaminetetraacetic acid (K_3_–EDTA) tube (BD Vacutainer, BD Biosciences, Oxford, UK) for preparation of serum and plasma respectively. Tubes for serum and plasma were centrifuged for 10 min at 2,000 × *g* and 4°C, and the supernatant was collected and stored at −80°C until analysis. PBMCs were isolated using density gradient centrifugation. Blood treated with sodium heparin was diluted 1:1 with sterile RPMI (Sigma-Aldrich, Gillingham, UK), layered onto Ficoll-Paque^TM^ plus (GE Healthcare, Buckinghamshire, UK) and centrifuged for 25 min at 500 × *g* and 21°C. PBMCs were washed in RPMI, by centrifuging for 10 min at 400 × *g* and 21°C and 7 min at 300 × *g* and 21°C. Platelets were removed by centrifuging for 7 min at 200 × *g* and 21°C. PBMCs were counted in Trypan blue (1.5% acetic acid) with a haemocytometer and a light microscope. PBMCs were resuspended in freezing mix (70% RPMI, 20% FCS and 10% DMSO), and frozen at −1°C/min in a freezing container (Mr Frosty, Nalgene) in a −80°C freezer.

### 2.5. Exercise interventions

#### 2.5.1. Partly-supervised exercise group

The partly supervised group undertook 2 supervised (laboratory-based treadmill walking and cycling) and 1 unsupervised session per week (e.g., outdoor walking) progressing from 35 to 50 min and 55% to 70% V˙O_2_max (Table 2). By week 7, the exercise prescription aligned with common physical activity recommendations (i.e., 150 min per week of moderate-vigorous intensity activity). During supervised laboratory sessions, intensity was confirmed and adjusted using indirect calorimetry. The intensity of unsupervised sessions was recorded using a chest-worn heart rate monitor (Wahoo Fitness, Atlanta, Georgia, USA).

**Table 2:** Exercise prescription.

	Week 1 & 2		Week 3 & 4		Week 5 & 6		Week 7 & 8	
	Duration (min)	Intensity (%V˙O_2_max)	Duration (min)	Intensity (%V˙O_2_max)	Duration (min)	Intensity (%V˙O_2_max)	Duration (min)	Intensity (%V˙O_2_max)
Partly-supervised group	105	55	120	60	135	65	150	70
Remotely-supported group	105	55	120	60	135	65	150	70
Breakdown								
Partly-supervised group	min		min		min		min	
Treadmill	20		25		30		35	
Bike	15		15		15		15	
Total after 1 session each week	35		40		45		50	
Total after 2 sessions each week	70		80		90		100	
Unsupervised walking	35		40		45		50	
Remotely-supported group	Bouts × min		Bouts × min		Bouts × min		Bouts × min	
Advice for achieving duration	3 × 35		3 × 40		3 × 45		3 × 50	
Minimum bout-length	≈11 × 10		12 × 10		≈14 × 10		15 × 10	

For the partly-supervised group, the exercise prescription was divided into 2 supervised and 1 unsupervised sessions per week. The supervised session took place in a laboratory and was further divided into treadmill walking and stationary cycle ergometer exercise. During supervised sessions, intensity was confirmed and adjusted using indirect calorimetry. Unsupervised sessions generally consisted of outdoor walking and intensity was prescribed using target heart rate thresholds corresponding to %V˙O_2_max.The remotely-supported group were asked to achieve the target duration and intensity (prescribed using target heart rate thresholds corresponding to %V˙O_2_max) across a given week in exercise bouts no shorter than 10 min bouts. The remotely-supported group were advised how they could breakdown their target into manageable bouts (e.g., 3 × 35 min walks = 105 min in week 1) and exercise generally consisted of outdoor walking.

#### 2.5.2. Remotely-supported exercise group

The remotely-supported group received a target for a total duration of exercise each week (e.g., outdoor walking) progressing in duration from 105 to 150 min and progressing in intensity from 55% to 70% V˙O_2_max (Table 2). By week 7, the exercise prescription aligned with common physical activity recommendations (i.e., 150 min per week of moderate-vigorous intensity activity). Participants were advised how they could breakdown their target into manageable bouts (e.g., 3 × 35 min walks = 105 min in week 1) and were instructed to accumulate their exercise with a minimum bout-length of 10 min. Intensity was checked by participants using heart-rate thresholds that corresponded to their cardiorespiratory fitness. Participants took part in a weekly 30 min telephone call to discuss the exercise they completed, as documented by a web-based data visualisation platform with data input from a wrist worn fitness tracker (Polar A370, Polar Electro, Kempele, Finland) that recorded accelerometery data and heart rate via photoplethysmography.

#### 2.5.3. Adherence

In the partly-supervised group, adherence was assessed by attendance and completion of each supervised session and participant verbal confirmation of completing the unsupervised session, verified via data interpretation of chest-worn heart-rate recording (Wahoo Fitness, Atlanta, Georgia, USA). In the remotely-supported group, adherence was assessed by participant verbal confirmation of reaching the weekly exercise target (duration and intensity) verified via data interpretation from wrist-worn heart rate-recording and accelerometery (Polar A370 fitness tracker (Polar Electro, Kempele, Finland).

### 2.6. Sample preparation prior to assays

PBMCs were cryopreserved for 6 ± 4 months and thawed rapidly in a 37°C water bath. PBMCs were washed in media (RPMI, 10% Foetal Calf Serum, and 1% penicillin-streptomycin) by centrifuging for 7 min at 300 × *g* and 21°C. If clumping was seen, PBMCs were treated with 0.3 µl of benzonase nuclease (250 U/µl, HC, Novogen) for 5 min, washed with media and centrifuged for 7 min at 300 × *g* and 21°C. PBMCs were counted, resuspended in media at a concentration of 2 million cells per ml and rested overnight for approximately 16 h in tubes that enabled gas exchange, at 37°C and 5% CO_2_. After resting overnight, PBMCs were counted and used for flow cytometry (see Section 2.7) and ELISpot assays (see Section 2.8).

### 2.7. Antibody panels, flow cytometry and data analysis

Two antibody panels were prepared; with 400,000 PBMCs added to a T cell panel and a B cell and Natural Killer (NK) cells panel. An additional isotype control panel was prepared to inform gating of T cell HLA-DR expression. PBMCs were stained with a Fixable Viability Stain (FVS) conjugated to v450 (Beckton Dickinson; Oxford, UK). The T cell panel included anti-CD3—PERCP clone SK7, anti-CD4—PE-Cy7 clone L200, anti-CD8—APC clone SK1, anti-CD45RA—FITC clone HI100, anti-CD127—APC-Cy7 clone A019D5, anti-HLA-DR—V500 clone G46-6, anti-CD27—PE clone M-T271 and anti-CD95—BV605 clone DX2. The B cell and NK cell panel included anti-CD3—PE-Cy7 clone SK7, anti-CD19—PERCP clone 4G7, anti-CD10—APC-Cy7 clone HI10a, anti-CD27—APC clone L128, anti-CD38—FITC clone HB7, anti-CD16—V500 clone 3G8 and anti-CD56—PE clone B159. The isotype control panel included HLA-DR—V500 isotype control (IgG2a, κ, clone G155-178) and anti-CD3—PERCP clone SK7. Antibodies were purchased from BD Biosciences (Beckton Dickinson; Oxford, UK) with the exception of anti-CD127—APC-Cy7, anti-CD95—BV605 and anti-CD10—APC-Cy7 (Biolegend, California, US).

Samples were analysed using a FACSAria III flow cytometer (Beckton Dickinson; Oxford, UK), within two hours of preparation. Voltages were optimised and maintained for all participants and all samples and acquisition flow rate was also maintained. Single-stained tubes containing positive and negative compensation beads (Beckton Dickinson; Oxford, UK) were used to perform compensation each day and calculated automatically (BD FACS DIVA^TM^, Beckton Dickinson; Oxford, UK). Approximately 70,000–100,000 events (T cell panel), 50,000–75,000 events (B cell panel) and 15,000 events (isotype control tube) were recorded from the lymphocyte gate. Data were analysed using Flowjo v10.7.1 (FlowJo. LLC, BD Biosiences, Beckton Dickinson; Oxford, UK).

After excluding doublets via forward-scatter (height) vs. forward-scatter (area) lymphocytes were gated via side-scatter vs. forward-scatter, and viability was assessed (typically >90%). T cells (CD3+) were divided into CD4+ and CD8+ and further defined as Naïve (NA: CD27+CD45RA+), Central Memory (CM: CD27+CD45RA−), Effector Memory (EM: CD27−CD45RA−) and Effector Memory expressing CD45RA (EMRA: CD27−CD45RA+). Using the isotype control, activated T cells were defined as HLA-DR+, and Median Fluorescence Intensity (MFI) was used to examine HLA-DR expression density. Stem cell like memory T cells (TSCMs) were identified as CD95+CD127+ from the CD4+ and CD8+ Naïve T cell pools (48–50). B cells (CD3−CD19+) were divided into Plasmablasts/Plasma cells (CD3−CD19+CD27+CD38+), Memory B cells (CD3−CD19+CD27+CD38−), Naïve B cells (CD3−CD19+CD27−CD10−) and Immature B cells (CD3−CD19+CD27−CD10+). Natural Killer cells (CD3−CD19−CD56+) were further defined into Effector (CD3−CD19−CD56+CD16+) and Regulatory cells (CD3−CD19−CD56+CD16−). Absolute cell counts were computed using the leukocyte differential determined in fresh whole K_3_–EDTA blood on the day of sampling (Sysmex Cell Counter Kx 21; Sysmex Europe, Germany) and the proportions of cells computed by the FlowJo software. The CD4+/CD8+ ratio was calculated to examine inverted or high ratios (<1 or >2.5), which have been linked to immunosenescence and chronic inflammation (51–53), and normal range was assumed between ≥1 and ≤2.5 (54).

### 2.8. ELISpot assays and data analysis

Enzyme-Linked ImmunoSpot (ELISPot) interferon-gamma (IFN-γ) assays were undertaken in a subset of participants with available samples (partly-supervised; *n* = 2; remotely-supported *n* = 8). Under sterile conditions, samples from both the pre- and post-intervention time points from the same participant were assayed on a single 96-well PVDF membrane plate, which had been activated for 30 s with 70% ethanol, washed three times with PBS, and incubated with an anti-IFN-γ antibody (7.5 µg/ml; clone 1D1K, Mabtech, Stockholm, Sweeden) for approximately 16 h at 4°C. After incubation, wells were washed three times with PBS, blocked with 100 µl of media for 1 h, and PBMCs added in 100 µl of media. Wells challenged with tumour-associated antigens contained 500,000 PBMCs, and wells challenged with virus antigens, or the positive control (anti-CD3 OKT3) or the negative control (DMSO) contained 300,000 PBMCs. Occasionally, for participants with low PBMC yields (*n* = 3) a lower number of PBMCs were added to wells challenged with tumour-associated antigens (minimum 300,000) and results adjusted accordingly.

The following antigens, depending on cell yields for each participant, were added to wells at 1 µg/ml (JPT peptide PepMixes; Berlin, Germany). Virus antigens; Cytomegalovirus (CMV) pp65, Varicella Zoster Virus (VZV) IE63 (*n* = 8 participants). Tumour-associated antigens: Mammaglobin, Survivin, Mucin-1 (*n* = 10 participants). Plates were incubated for approximately 16 h at 37°C and 5% CO_2_. After incubation, plates were washed eight times with PBS 0.05% TWEEN 20 (200 µl per well) and incubated for three hours with an anti-IFN-γ antibody (1 µg/ml; clone 7-B6-1, Mabtech, Stockholm, Sweeden). Plates were washed eight times with PBS 0.05% TWEEN 20 (200 µl per well) and wells incubated with Streptavidin-Alkaline Phosphatase (diluted 1:1,000; Mabtech, Stockholm, Sweeden) for 1.5 h. Plates were washed eight times with PBS 0.05% TWEEN 20 (200 µl per well) and a further three times with PBS only, before a chromogen substrate (Alkaline phosphatase conjugate substrate kit, Bio-Rad Laboratories Inc.; Watford, UK) was added following manufacturer's instructions. The reaction was stopped after 45–60 min by washing the plate with tap water. The plate was left to dry for at least 24 h before counting spots on an AID classic ELISpot reader [AID software, Autoimmun Diagnostika GmbH (AID), Strassberg, Germany]. Camera and counting settings were optimized and maintained for all samples and all participants. Data were expressed as spots per million PBMCs.

### 2.9. Assessment of cytomegalovirus (CMV) serostatus

CMV-specific IgG antibodies were measured in serum using enzyme-linked immunosorbent assays (ELISAs) according to manufacturer instructions (ENZY-WELL, Diesse Diagnostica, Italy). A SPECTROstar Nano plate reader (BMG Labtech Ltd., UK) was used and absorbance was determined at 450 nm. Values were blank corrected, and a 4-parameter curve was used to calculate concentrations. CMV+ was considered to be >1.2 IU/ml, and CMV− was <0.8 IU/ml. Inter and intra assay variation was 1.74% and <5.2% respectively.

### 2.10. Statistical analysis

Data were examined for normal distribution using descriptive statistics, Shapiro Wilks and Kolmogorov-Smirnov tests. Non-normally distributed data were log10 transformed. Pre- and post-intervention data were examined using repeated measures analyses of variance (ANOVAs) controlling for baseline values and analysing the effect of group. Subsequent post-hoc univariate ANOVAs were conducted for each group separately when appropriate. Effect sizes were reported as eta squared (*η*^2^), where, *η*^2 ^= 0.14 is considered a large effect, *η*^2 ^= 0.06 a medium effect and *η*^2 ^= 0.01 a small effect (55). Statistical significance was considered at *p* < 0.05. Statistical analyses were performed with SPSS v27.0.1.0 (IBM Corp.; New York, US) and figures were created with GraphPad Prism v9.0.0 for Windows (GraphPad Software; California, US).

## 3. Results

### 3.1. Body composition and cardiorespiratory fitness did not change with 8 weeks of exercise training, but some measures of functional fitness were improved

Intervention adherence was generally higher in the partly-supervised group compared to the remotely-supported group (88 ± 8% vs. 70 ± 25% of sessions completed) but this difference was not statistically significant (*F*_(1,19) _= 4.150, *p* = 0.056, *η*^2 ^= 0.187). All sessions were attempted by all participants (i.e., no sessions were missed), therefore the lack of adherence reflects failure to achieve the prescribed duration and intensity of sessions. Body composition and cardiorespiratory fitness did not change with eight weeks of exercise training (main effect: *F*_(1,17) _< 1.144, *p* > 0.300, *η*^2 ^< 0.063) (Tables 3, 4). However, distance walked in the six minute walk test increased (main effect: *F*_(1,17) _= 12.970, *p* = 0.002, *η*^2 ^= 0.433) driven by a statistically significant increase in the partly-supervised group, which was 5% greater compared to the remotely-supported group (one-way ANOVA for the partly-supervised group: *F*_(1,8) _= 14.020, *p* = 0.006, *η*^2 ^= 0.637) (Table 4). In addition, the sit to stand score improved (main effect: *F*_(1,17) _= 11.368, *p* = 0.004, *η*^2 ^= 0.401), with improvements in both groups (partly-supervised; *F*_(1,8) _= 4.301, *p* = 0.072, *η*^2 ^= 0.350; remotely-supported; *F*_(1,8) _= 6.830, *p* = 0.031, *η*^2 ^= 0.461). There was also a trend for an improvement for the get up and go test (main effect: *F*_(1,17) _= 3.417, *p* = 0.082, *η*^2 ^= 0.167). There were no statistically significant time × group interaction effects.

**Table 3:** Body composition.

	Pre-intervention	Post-intervention	Main effect of time	Time × group interaction effect
Body mass (kg)				
Whole group	66.87 ± 7.90	67.21 ± 8.39	*F*_(1,17) _= 0.820, *p* = 0.378, *η*^2 ^= 0.046	*F*_(1,17) _= 0.002, *p* = 0.962, *η*^2 ^= 0.000
Partly-supervised	67.05 ± 8.25	67.38 ± 8.50	*F*_(1,8) _= 0.427, *p* = 0.532, *η*^2 ^= 0.051	
Remotely-supported	66.68 ± 7.97	67.03 ± 8.73	*F*_(1,8) _= 0.639, *p* = 0.560, *η*^2 ^= 0.044	
BMI (kg/m^2^)				
Whole group	25.38 ± 2.98	25.53 ± 3.13	*F*_(1,17) _= 0.676, *p* = 0.422, *η*^2 ^= 0.038	*F*_(1,17) _= 0.002, *p* = 0.966, *η*^2 ^= 0.000
Partly-supervised	25.84 ± 3.31	26.01 ± 3.45	*F*_(1,8) _= 0.285, *p* = 0.608, *η*^2 ^= 0.034	
Remotely-supported	24.91 ± 2.70	25.05 ± 2.87	*F*_(1,8) _= 0.399, *p* = 0.545, *η*^2 ^= 0.047	
Body fat (%)				
Whole group	36.18 ± 5.88	36.82 ± 5.49	*F*_(1,17) _= 0.458, *p* = 0.507, *η*^2 ^= 0.026	*F*_(1,17) _= 0.176, *p* = 0.680, *η*^2 ^= 0.010
Partly-supervised	37.32 ± 4.50	37.16 ± 4.04	*F*_(1,8) _= 0.140, *p* = 0.718, *η*^2 ^= 0.017	
Remotely-supported	35.03 ± 7.06	36.48 ± 6.86	*F*_(1,8) _= 0.529, *p* = 0.488, *η*^2 ^= 0.062	
Lean mass (kg)				
Whole group	41.27 ± 4.01	41.52 ± 4.12	*F*_(1,17) _= 1.144, *p* = 0.300, *η*^2 ^= 0.063	*F*_(1,17) _= 0.004, *p* = 0.952, *η*^2 ^= 0.000
Partly-supervised	41.31 ± 4.22	41.55 ± 4.34	*F*_(1,8) _= 0.354, *p* = 0.568, *η*^2 ^= 0.042	
Remotely-supported	41.22 ± 4.03	41.49 ± 4.12	*F*_(1,8) _= 0.928, *p* = 0.364, *η*^2 ^= 0.104	
Fat mass (kg)				
Whole group	24.69 ± 5.61	24.80 ± 5.90	*F*_(1,17) _= 0.138, *p* = 0.714, *η*^2 ^= 0.008	*F*_(1,17) _= 0.271, *p* = 0.609, *η*^2 ^= 0.016
Partly-supervised	24.92 ± 5.34	25.18 ± 5.37	*F*_(1,8) _= 0.387, *p* = 0.551, *η*^2 ^= 0.046	
Remotely-supported	24.46 ± 6.15	24.41 ± 6.66	*F*_(1,8) _= 0.016, *p* = 0.904, *η*^2 ^= 0.002	
Fat mass index (kg/m^2^)				
Whole group	9.39 ± 2.22	9.44 ± 2.30	*F*_(1,17) _= 0.128, *p* = 0.725, *η*^2 ^= 0.007	*F*_(1,17) _= 0.291, *p* = 0.597, *η*^2 ^= 0.017
Partly-supervised	9.63 ± 2.21	9.74 ± 2.21	*F*_(1,8) _= 0.330, *p* = 0.581, *η*^2 ^= 0.040	
Remotely-supported	9.16 ± 2.33	9.14 ± 2.47	*F*_(1,8) _= 0.025, *p* = 0.879, *η*^2 ^= 0.003	

Data are mean ± standard deviation (SD) for breast cancer survivors, as a whole group (*n* = 20), and also as part of the two different study groups: partly-supervised group (*n* = 10) and remotely-supported group (*n* = 10). Repeated measures ANOVA were performed in raw data for variables that were normally distributed (body mass, BMI, lean mass, fat mass, fat mass index), and in log10 transformed data for variables that deviated significantly from the normal distribution (body fat percentage). Statistical significance was considered as *p* < 0.05. Main effect of time indicated as *p < 0.05, **p < 0.01 or ***p < 0.001, time × group effect indicated as ^†^*p* < 0.05, ^††^*p* < 0.01 or ^†††^*p* < 0.001. BMI, body mass index

**Table 4:** Cardiorespiratory fitness and functional measurements.

	Pre-intervention	Post-intervention	Main effect of time	Time × group interaction effect
V˙O_2_max (ml kg^−1^ min^−1^)				
Whole group	29.07 ± 5.55	28.25 ± 5.28	*F*_(1,17) _= 0.680, *p* = 0.421, *η*^2 ^= 0.038	*F*_(1,17) _= 1.029, *p* = 0.325, *η*^2 ^= 0.057
Partly-supervised	28.11 ± 4.83	28.63 ± 5.49	*F*_(1,8) _= 0.193, *p* = 0.672, *η*^2 ^= 0.024	
Remotely-supported	30.03 ± 6.30	27.87 ± 5.34	*F*_(1,8) _= 2.467, *p* = 0.155, *η*^2 ^= 0.236	
Six minute walk (m)				
Whole group	478.0 ± 42.4	508.9 ± 51.3**	*F*_(1,17) _= 12.970, *p* = 0.002, *η*^2 ^= 0.433	*F*_(1,17) _= 1.783, *p* = 0.199, *η*^2 ^= 0.095
Partly-supervised	473.6 ± 36.6	516.6 ± 38.9**	*F*_(1,8) _= 14.020, *p* = 0.006, *η*^2 ^= 0.637	
Remotely-supported	482.5 ± 49.2	501.1 ± 62.6	*F*_(1,8) _= 2.282, *p* = 0.169, *η*^2 ^= 0.222	
Sit to stand (reps)				
Whole group	16 ± 4	19 ± 5**	*F*_(1,17) _= 11.368, *p* = 0.004, *η*^2 ^= 0.401	*F*_(1,17) _= 0.000, *p* = 0.998, *η*^2 ^= 0.000
Partly-supervised	17 ± 4	19 ± 6	*F*_(1,8) _= 4.301, *p* = 0.072, *η*^2 ^= 0.350	
Remotely-supported	16 ± 3	18 ± 5*	*F*_(1,8) _= 6.830, *p* = 0.031, *η*^2 ^= 0.461	
Get up and go (s)				
Whole group	5.12 ± 0.67	4.84 ± 0.66	*F*_(1,17) _= 3.417, *p* = 0.082, *η*^2 ^= 0.167	*F*_(1,17) _= 0.044, *p* = 0.836, *η*^2 ^= 0.003
Partly-supervised	4.99 ± 0.84	4.85 ± 0.67	*F*_(1,8) _= 0.402, *p* = 0.544, *η*^2 ^= 0.048	
Remotely-supported	5.26 ± 0.47	4.83 ± 0.67	*F*_(1,8) _= 3.916, *p* = 0.083, *η*^2 ^= 0.329	

Data are mean ± standard deviation (SD) for breast cancer survivors, as a whole group (*n* = *20*), and also as part of the two different study groups: partly-supervised group (*n* = 10) and remotely-supported group (*n* = 10). Repeated measures ANOVA were performed in raw data for variables that were normally distributed (six-minutes’ walk and get up and go), and in log10 transformed data for variables that deviated significantly from the normal distribution (V˙O_2_max and sit to stand). Statistical significance was considered as *p* < 0.05. Main effect of time indicated as *p < 0.05, **p < 0.01 or ***p < 0.001, time × group effect indicated as ^†^*p* < 0.05, ^††^*p* < 0.01 or ^†††^*p* < 0.001. reps = repetitions, V˙O_2_max = cardiorespiratory fitness.

### 3.2. Total leukocyte counts did not change with 8 weeks of exercise training and CMV-specific IgG declined

There were no significant differences in total leukocytes, lymphocytes, monocytes and neutrophils with eight weeks of exercise training (main effect: *F*_(1,17) _< 0.925, *p* > 0.350, *η*^2 ^> 0.052), and each group responded similarly (interaction effect: *F*_(1,17) _< 2.629, *p* > 0.123, *η*^2 ^< 0.134) (Table 5). Among CMV + participants, CMV-specific IgG significantly decreased (*F*_(1,6) _= 6.602, *p* = 0.042, *η*^2 ^= 0.524) but each group responded similarly (interaction effect: *F*_(1,6) _= 1.262, *p* = 0.304, *η*^2 ^= 0.174). However, the magnitude of the decrease in the partly-supervised group was 7% greater compared to the remotely-supported group and was closer to statistical significance (one-way ANOVA for the partly-supervised group: *F*_(1,3) _= 9.206, *p* = 0.056, *η*^2 ^= 0.754).

**Table 5:** Leukocyte differential and CMV serostatus.

Cell count (×10^9^/L)	Pre-intervention	Post-intervention	Main effect of time controlled for baseline	Time × group interaction effect
Lymphocytes				
Whole group	1.46 ± 0.40	1.41 ± 0.34	*F*_(1,17) _= 0.925, *p* = 0.350, *η*^2 ^= 0.052	*F*_(1,17) _= 0.220, *p* = 0.645, *η*^2 ^= 0.013
Partly-supervised	1.52 ± 0.36	1.47 ± 0.32	*F*_(1,8) _= 0.387, *p* = 0.551, *η*^2 ^= 0.046	
Remotely-supported	1.39 ± 0.45	1.34 ± 0.36	*F*_(1,8) _= 0.503, *p* = 0.498, *η*^2 ^= 0.059	
Monocytes				
Whole group	0.44 ± 0.19	0.45 ± 0.19	*F*_(1,17) _= 0.087, *p* = 0.772, *η*^2 ^= 0.005	*F*_(1,17) _= 2.629, *p* = 0.123, *η*^2 ^= 0.134
Partly-supervised	0.51 ± 0.20	0.54 ± 0.14	*F*_(1,8) _= 0.659, *p* = 0.441, *η*^2 ^= 0.076	
Remotely-supported	0.36 ± 0.14	0.35 ± 0.19	*F*_(1,8) _= 0.028, *p* = 0.870, *η*^2 ^= 0.004	
Neutrophils				
Whole group	3.15 ± 1.15	3.10 ± 1.08	*F*_(1,17) _= 0.084, *p* = 0.776, *η*^2 ^= 0.005	*F*_(1,17) _= 1.466, *p* = 0.242, *η*^2 ^= 0.079
Partly-supervised	3.61 ± 1.36	3.16 ± 1.08	*F*_(1,8) _= 3.214, *p* = 0.111, *η*^2 ^= 0.287	
Remotely-supported	2.69 ± 0.68	3.03 ± 1.14	*F*_(1,8) _= 1.523, *p* = 0.252, *η*^2 ^= 0.160	
CMV IgG (IU/ml)^2^				
Whole group (*n* = 9)	18.11 ± 4.37	16.59 ± 4.47*	*F*_(1,6) _= 6.602, *p* = 0.042, *η*^2 ^= 0.524	*F*_(1,6) _= 1.262, *p* = 0.304, *η*^2 ^= 0.174
Partly-supervised (*n* = 5)	16.15 ± 4.89	14.23 ± 3.92	*F*_(1,3) _= 9.206, *p* = 0.056, *η*^2 ^= 0.754	
Remotely-supported (*n* = 4)	20.57 ± 2.17	19.53 ± 3.47	*F*_(1,2) _= 2.236, *p* = 0.273, *η*^2 ^= 0.528	

Data are mean standard ± deviation (SD) for breast cancer survivors, as a whole group (*n* = 20), and also as part of the two different study groups: partly-supervised group (*n* = 10) and remotely-supported (*n* = 10). Repeated measures ANOVAs were performed in raw data, as this was normally distributed. Monocytes refer to the “Mixed cells’ fraction from an automated haematology analyser: <10% correspond to basophils and eosinophils. ^2^CMV IgG concentration for CMV seropositive individuals (partly-supervised group: *n* = 5; remotely-supported group: *n* = 4). Statistical significance was considered as *p* < 0.05. Main effect of time indicated as *p < 0.05, **p < 0.01 or ***p < 0.001, time × group effect indicated as ^†^*p* < 0.05, ^††^*p* < 0.01 or ^†††^*p* < 0.001. CMV, Cytomegalovirus, IgG, Immunoglobulin G.

### 3.3. T cell subset counts were largely unchanged with 8 weeks of exercise training, but CD4+ EMRA T cells declined

**Figure 1:**
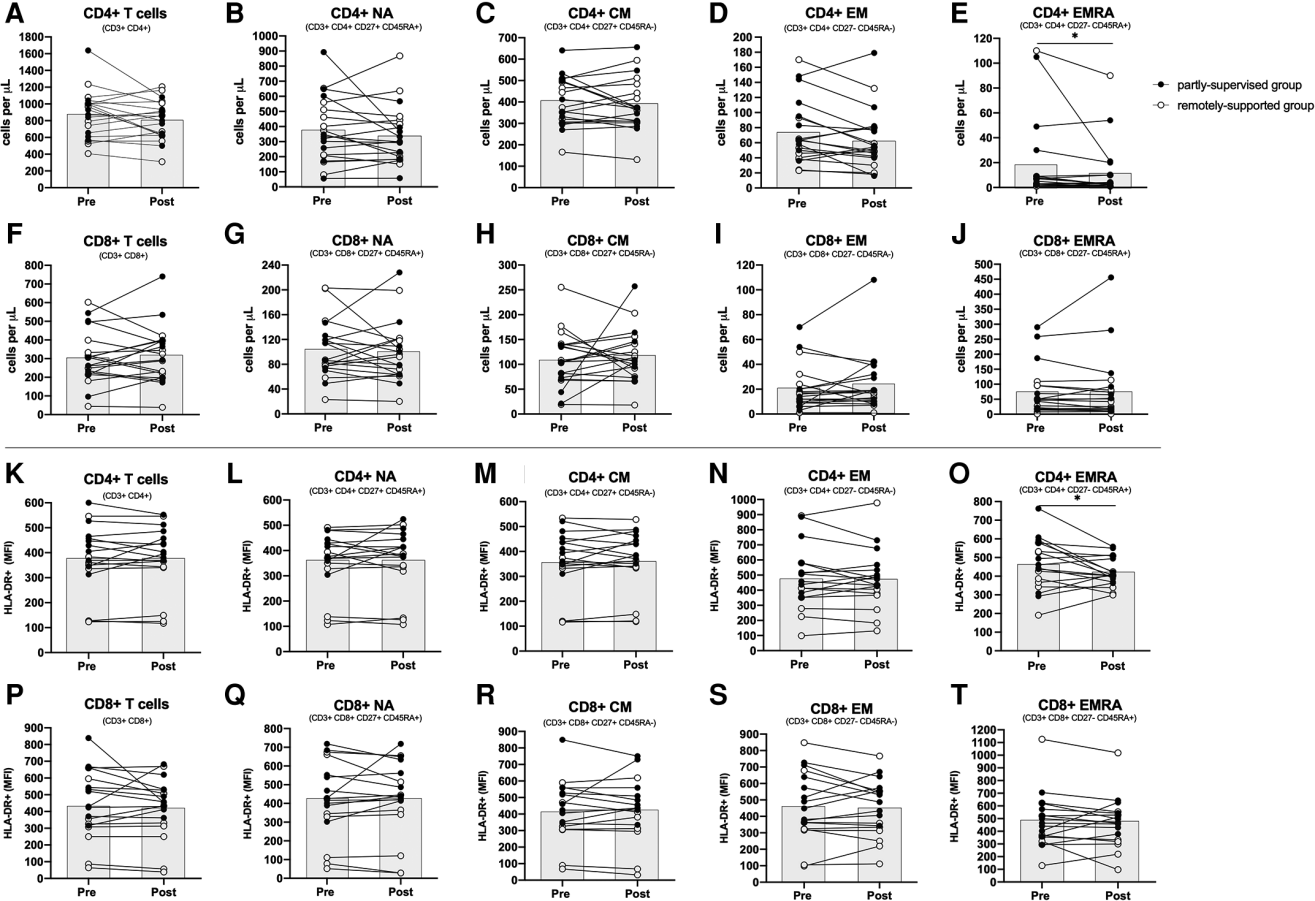
Absolute counts and HLA-DR expression density among CD4+ and CD8+ T cell subsets. Absolute counts (**panels A–J**) and HLA-DR expression density expressed as median fluorescence intensity (MFI) (**panels K–T**) for CD4+ and CD8+ T cell subsets: Naïve (NA), Central Memory (CM), Effector Memory (EM) and Effector Memory expressing CD45RA (EMRA). (*n* = 20 for absolute counts; *n* = 18 for activation). Data are displayed for each participant and from both study groups: partly-supervised group (black data points, *n* = 10 for absolute counts and activation) and remotely-supported group (white data points, *n* = 10 for absolute counts; *n* = 8 for activation). Datapoints from the same participant have been joined with lines and the group mean is displayed with the grey bars. Statistical significance from repeated measures ANOVAs using log10 transformed data in the whole group is shown. Statistical significance was considered as *p* < 0.05 (*p < 0.05).

CD4+ and CD8+ T cells and most of their sub-types did not change with eight weeks of exercise training (main effect: *F*_(1,17) _< 2.871, *p* > 0.108, *η*^2 ^< 0.144) (see Figure 1, panels A–J). However, there were significantly fewer CD4+ EMRA T cells after training across all participants (main effect: *F*_(1,17) _= 5.985, *p* = 0.026, *η*^2 ^= 0.260). Both groups responded similarly (interaction effect: *F*_(1,17) _= 2.076, *p* = 0.168, *η*^2 ^= 0.109), although the partly-supervised group decreased 30% more compared to the remotely-supported group (one-way ANOVA for the partly-supervised group: *F*_(1,8) _= 4.849, *p* = 0.059, *η*^2 ^= 0.377). There were also fewer CD4+ EM cells after training across all participants but this did not reach statistical significance (main effect: *F*_(1,17) _= 4.399, *p* = 0.051, *η*^2 ^= 0.206). Although there was no main effect for CD8+ EM, there was a statistically significant group × time interaction effect (interaction effect: *F*_(1,17) _= 7.258, *p* = 0.015, *η*^2 ^= 0.299). When these analyses were repeated in both study groups separately, the partly-supervised group showed an increase in the CD8+ EM counts following training that was close to being statistically significant (Pre-intervention: 22 ± 22 cells/µl vs. Post-intervention: 32 ± 29 cells/µl; one-way ANOVA: *F*_(1,8) _= 4.682, *p* = 0.062, *η*^2 ^= 0.369).

### 3.4. T cell subset activation was largely unchanged with 8 weeks of exercise training, but CD4+ EMRA T cell activation declined

Overall, after eight weeks of exercise training across groups, there were no changes to basal unstimulated T cell activation, assessed by HLA-DR expression density (*F*_(1,15) _< 1.496, *p* > 0.240, *η*^2 ^< 0.091). However, for CD4+ EMRA T cells, activation significantly declined following training across all participants (main effect: *F*_(1,15) _= 7.003, *p* = 0.018, *η*^2 ^= 0.318). Both groups responded similarly (interaction effect: *F*_(1,15) _= 1.413, *p* = 0.253, *η*^2 ^= 0.086) (see Figure 1, panels K–T). The proportion of T cells and their subtypes expressing the activation marker HLA-DR was also assessed as an alternative measure of activation, but this did not change following training (*F*_(1,15) _< 0.841, *p* > 0.374, *η*^2 ^< 0.053, **data not shown**).

### 3.5. Partly-supervised exercise significantly reduced the CD4+/CD8+ ratio towards more normal values

Overall across groups, there was no change to the CD4+/CD8+ ratio following eight weeks of exercise training (main effect: *F*_(1,17) _= 2.885, *p* = 0.108, *η*^2 ^= 0.145) (see Table 6), but there was a statistically significant group × time interaction effect (interaction effect: *F*_(1,17) _= 6.525, *p* = 0.021, *η*^2 ^= 0.277). When these analyses were repeated for both study groups separately, the partly-supervised group showed a decrease in the CD4+/CD8+ ratio following training that was statistically significant (Pre-intervention: 3.90 ± 2.98 vs. Post-intervention: 2.54 ± 1.29; one-way ANOVA: *F*_(1,8) _= 13.795, *p* = 0.006, *η*^2 ^= 0.633).

**Table 6:** CD4+/CD8+ ratio and absolute counts for TSCMs, B cell and NK cell subsets.

Cells per µl	Pre-intervention	Post-intervention	Main effect of time controlled for baseline	Time × group interaction effect
CD4+/CD8+ ratio				
Whole group	3.85 ± 3.03	3.40 ± 2.89^†^	*F*_(1,17) _= 2.885, *p* = 0.108, *η*^2 ^= 0.145	*F*_(1,17) _= 6.525, *p* = 0.021, *η*^2 ^= 0.277
Partly-supervised	3.90 ± 2.98	2.54 ± 1.29**	*F*_(1,8) _= 13.795, *p* = 0.006, *η*^2 ^= 0.633	
Remotely-monitored	3.80 ± 3.24	4.26 ± 3.79	*F*_(1,8) _= 0.780, *p* = 0.403, *η*^2 ^= 0.089	
CD4+ TSCMs				
Whole group	2.35 ± 1.04	2.60 ± 1.60	*F*_(1,17) _= 1.017, *p* = 0.327, *η*^2 ^= 0.056	*F*_(1,17) _= 0.226, *p* = 0.641, *η*^2 ^= 0.013
Partly-supervised	2.30 ± 1.16	2.40 ± 1.27	*F*_(1,8) _= 0.191, *p* = 0.674, *η*^2 ^= 0.023	
Remotely-supported	2.40 ± 0.97	2.80 ± 1.93	*F*_(1,8) _= 0.832, *p* = 0.388, *η*^2 ^= 0.094	
CD8+ TSCMs				
Whole group	0.30 ± 0.47	0.35 ± 0.49	*F*_(1,17) _= 0.230, *p* = 0.637, *η*^2 ^= 0.013	*F*_(1,17) _= 0.002, *p* = 0.963, *η*^2 ^= 0.000
Partly-supervised	0.40 ± 0.52	0.40 ± 0.52	*F*_(1,8) _= 0.000, *p* = 1.000, *η*^2 ^= 0.000	
Remotely-supported	0.20 ± 0.42	0.30 ± 0.48	*F*_(1,8) _= 0.400, *p* = 0.545, *η*^2 ^= 0.048	
CD19+ total (B cells)				
Whole group	28.30 ± 28.51	31.20 ± 28.68	*F*_(1,17) _= 0.528, *p* = 0.477, *η*^2 ^= 0.030	*F*_(1,17) _= 0.012, *p* = 0.914, *η*^2 ^= 0.001
Partly-supervised	21.70 ± 11.20	27.90 ± 27.27	*F*_(1,8) _= 0.334, *p* = 0.579, *η*^2 ^= 0.040	
Remotely-supported	34.90 ± 38.66	34.50 ± 31.12	*F*_(1,8) _= 0.163, *p* = 0.697, *η*^2 ^= 0.020	
Plasmablasts				
Whole group	1.20 ± 1.24	1.10 ± 1.12	*F*_(1,17) _= 0.865, *p* = 0.365, *η*^2 ^= 0.048	*F*_(1,17) _= 3.743, *p* = 0.070, *η*^2 ^= 0.180
Partly-supervised	1.10 ± 1.52	0.80 ± 1.32	*F*_(1,8) _= 2.688, *p* = 0.140, *η*^2 ^= 0.251	
Remotely-supported	1.30 ± 0.95	1.40 ± 0.84	*F*_(1,8) _= 0.689, *p* = 0.431, *η*^2 ^= 0.079	
Memory B cells				
Whole group	6.00 ± 9.67	5.95 ± 6.52	*F*_(1,17) _= 0.273, *p* = 0.608, *η*^2 ^= 0.016	*F*_(1,17) _= 0.122, *p* = 0.731, *η*^2 ^= 0.007
Partly-supervised	2.20 ± 2.82	3.70 ± 6.34	*F*_(1,8) _= 0.366, *p* = 0.562, *η*^2 ^= 0.044	
Remotely-supported	9.80 ± 12.54	8.20 ± 6.18	*F*_(1,8) _= 0.007, *p* = 0.937, *η*^2 ^= 0.001	
Immature B cells				
Whole group	1.25 ± 1.59	1.35 ± 1.81	*F*_(1,17) _= 0.000, *p* = 1.000, *η*^2 ^= 0.000	*F*_(1,17) _= 0.002, *p* = 0.963, *η*^2 ^= 0.001
Partly-supervised	0.90 ± 0.74	1.30 ± 2.11	*F*_(1,8) _= 0.021, *p* = 0.889, *η*^2 ^= 0.003	
Remotely-supported	1.60 ± 2.12	1.40 ± 1.58	*F*_(1,8) _= 0.073, *p* = 0.794, *η*^2 ^= 0.009	
Naive B cells				
Whole group	19.65 ± 17.54	22.55 ± 23.75	*F*_(1,17) _= 0.075, *p* = 0.788, *η*^2 ^= 0.004	*F*_(1,17) _= 0.744, *p* = 0.400, *η*^2 ^= 0.042
Partly-supervised	17.00 ± 8.33	21.60 ± 22.19	*F*_(1,8) _= 0.504, *p* = 0.498, *η*^2 ^= 0.059	
Remotely-supported	22.30 ± 23.76	23.50 ± 26.40	*F*_(1,8) _= 0.112, *p* = 0.746, *η*^2 ^= 0.014	
CD56+ total (NK cells)				
Whole group	117.65 ± 78.47	120.00 ± 70.26^†^	*F*_(1,17) _= 1.278, *p* = 0.274, *η*^2 ^= 0.070	*F*_(1,17) _= 4.473, *p* = 0.049, *η*^2 ^= 0.208
Partly-supervised	121.00 ± 102.81	136.70 ± 89.65	*F*_(1,8) _= 2.685, *p* = 0.140, *η*^2 ^= 0.251	
Remotely-supported	114.30 ± 49.01	103.30 ± 42.02	*F*_(1,8) _= 1.359, *p* = 0.277, *η*^2 ^= 0.145	
CD16+ Effector cells				
Whole group	98.25 ± 75.71	100.35 ± 67.80	*F*_(1,17) _= 1.357, *p* = 0.260, *η*^2 ^= 0.074	*F*_(1,17) _= 3.647, *p* = 0.073, *η*^2 ^= 0.177
Partly-supervised	105.10 ± 99.44	116.30 ± 86.79	*F*_(1,8) _= 2.234, *p* = 0.173, *η*^2 ^= 0.218	
Remotely-supported	91.40 ± 45.91	84.40 ± 40.09	*F*_(1,8) _= 0.555, *p* = 0.478, *η*^2 ^= 0.065	
CD16− Regulatory cells				
Whole group	19.30 ± 10.37	19.60 ± 8.99^†^	*F*_(1,17) _= 1.566, *p* = 0.228, *η*^2 ^= 0.084	*F*_(1,17) _= 7.755, *p* = 0.013, *η*^2 ^= 0.313
Partly-supervised	16.00 ± 7.83	20.50 ± 10.28**	*F*_(1,8) _= 10.667, *p* = 0.011, *η*^2 ^= 0.571	
Remotely-monitored	22.60 ± 11.89	18.70 ± 7.95	*F*_(1,8) _= 3.742, *p* = 0.089, *η*^2 ^= 0.319	

Data are mean standard ± deviation (SD) for breast cancer survivors, as a whole group (*n* = 20), and also as part of the two different study groups: partly-supervised group (*n* = 10) and remotely-supported group (*n* = 10). Repeated measures ANOVAs were performed in log10 transformed data (or log10 + 1 in the case of CD4+ and CD8+ TSCMs, Plasmablasts, Memory B cells and Immature B cells) as the majority of variables were not normally distributed. Statistical significance was considered as *p* < 0.05. Main effect of time indicated as *p < 0.05, **p < 0.01 or ***p < 0.001, time × group effect indicated as ^†^*p* < 0.05, ^††^*p* < 0.01 or ^†††^*p* < 0.001.

Pre-intervention, across all participants, 35% had a normal CD4+/CD8+ ratio (≥1 or ≤2.5) and 65% had a high ratio (>2.5). Post-intervention, the percentage of participants with a normal ratio increased to 45% and the percentage of participants with a high ratio decreased to 55% (Pearson Chi square: 0.417 p_2−sided _= 0.748, **data not shown**). When groups were examined separately, each group exhibited divergent responses. In the remotely-supported group, 30% of participants had a normal ratio and 70% had a high ratio pre-intervention and this distribution was unchanged post-intervention (Pearson Chi square: 0.000 p_2−sided _= 1.000). However, in the partly-supervised group, 40% of the participants had a normal ratio and 60% had a high ratio pre-intervention, but this distribution inverted post-intervention: 60% of partly-supervised participants had a normal ratio and 40% had a high ratio (Pearson Chi square: 0.800 p_2−sided _= 0.656).

### 3.6. TSCM, B cell and NK cell counts were largely unchanged by 8 weeks of exercise training

The counts of CD4+ and CD8+ TSCMs did not change following eight weeks of exercise training (main effects: *F*_(1,17) _< 1.017, *p* > 0.327, *η*^2 ^< 0.056) (see Table 6) and there were no statistically significant group × time interaction effects (*F*_(1,17) _< 0.226, *p* > 0.641, *η*^2 ^< 0.013). Furthermore, the counts of B cells and their subtypes (Plasmablasts/Plasma cells, Memory B cells, Immature B cells and Naïve B cells) did not change with training (main effects: *F*_(1,17) _< 0.865, *p* > 0.365, *η*^2 ^< 0.048) and there were no statistically significant group × time interaction effects (*F*_(1,17) _< 3.743, *p* > 0.070, *η*^2 ^< 0.180). In addition, the counts of NK cells and NK cell subtypes (CD16+ effector and CD16− regulatory cells) did not significantly change following training (main effects: *F*_(1,17) _< 1.566, *p* > 0.228, *η*^2 ^< 0.084). However, there was a statistically significant group × time interaction effect for total NK cells (*F*_(1,17) _= 4.473, *p* = 0.049, *η*^2 ^= 0.208) and CD16− Regulatory cells (*F*_(1,17) _= 7.755, *p* = 0.013, *η*^2 ^= 0.313) and the group × time interaction effect for CD16+ effector cells was close to significance (*F*_(1,17) _= 3.647, *p* = 0.073, *η*^2 ^= 0.177). When these analyses were repeated for both study groups separately, there was a statistically significant increase in CD16− Regulatory cells in the partly-supervised group with training (Pre-intervention: 16 ± 8 cells/µl vs. Post-intervention: 21 ± 10 cells/µl; one-way ANOVA: *F*_(1,8) _= 10.667, *p* = 0.011, *η*^2 ^= 0.571).

### 3.7. T cell IFN-γ production in response to stimulation with virus and tumour associated antigens

**Figure 2:**
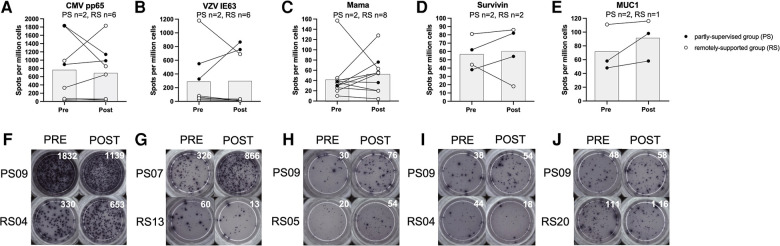
T cell interferon-gamma production in response to stimulation with virus and tumour-associated antigens. Data is expressed as spots per million cells and reflects IFN-γ production from T cells as a result of antigen recognition (panels A-E). Individual timepoints for the same participant have been joined with lines and the group mean is displayed with the grey bars. Repeated measures ANOVAs using log10 transformed data were performed for CMV pp65, VZV IE63 and Mama (Mammaglobin). Data for Survivin and MUC1 (Mucin-1) is presented for qualitative interpretation, as statistical comparisons were not appropriate due to the small sample size. Statistical significance was considered as *p* < 0.05. Representative examples of ELISPot wells from individual participants from each study group have been shown for each antigen, pre vs. post intervention (panels F-J).

T cell IFN-γ production in response to stimulation with VZV IE63 did not significantly change with eight weeks of exercise training (*n* = 8, main effect: *F*_(1,5) _= 0.228, *p* = 0.653, *η*^2 ^= 0.044) and there was no statistically significant group × time interaction effect (interaction effect: *F*_(1,5) _= 5.018, *p* = 0.075, *η*^2 ^= 0.501). However, this VZV-specific response increased among the partly-supervised group (Pre-intervention; 438 ± 158 spots vs. post-intervention; 811 ± 78 spots) and decreased among the remotely-supported group with training (Pre-intervention; 235 ± 463 spots vs. post-intervention; 129 ± 274 spots) (see Figure 2).

T cell IFN-γ production in response to stimulation with CMV pp65 did not significantly change with eight weeks of exercise training (*n* = 8, main effect: *F*_(1,5) _= 0.288, *p* = 0.615, *η*^2 ^= 0.054), and there was no significant group × time interaction effect (*F*_(1,5) _= 0.001, *p* = 0.982, *η*^2 ^= 0.000). However, this response decreased among the partly-supervised group (Pre-intervention; 1,364 ± 662 spots vs. post-intervention; 1,063 ± 108 spots) and increased among the remotely-supported group with training (Pre-intervention; 547 ± 728 spots vs. post-intervention; 581 ± 706 spots).

T cell IFN-γ production in response to stimulation with Mammaglobin did not significantly change with eight weeks of exercise training (*n* = 10, main effect: *F*_(1,7) _= 0.469, *p* = 0.515, *η*^2 ^= 0.063) and there was no significant group × time interaction effect (*F*_(1,7) _= 0.259, *p* = 0.626, *η*^2 ^= 0.036). However, this response increased among the partly-supervised group (Pre-intervention; 34 ± 6 spots vs. post-intervention; 56 ± 28 spots) and also the remotely-supported group with training (Pre-intervention; 46 ± 46 spots vs. post intervention; 50 ± 38 spots).

T cell IFN-γ production in response to stimulation with Survivin and Mucin-1 were not examined statistically due to a small sample size, but data are shown for qualitative comparison and interpretation. However, there was a similar pattern of response as shown for Mammaglobin. For Survivin, the response increased with eight weeks of exercise training in the partly-supervised group (Pre-intervention; 50 ± 17 spots vs. post-intervention; 68 ± 20 spots), and it decreased in the remotely-supported group (Pre-intervention; 63 ± 26 spots vs. post-intervention; 52 ± 48 spots). For Mucin-1, the response increased in the partly-supervised group (Pre-intervention; 53 ± 7 spots vs. post-intervention 78 ± 28 spots), and was largely unchanged in the remotely-supported group (*n* = 1, Pre-intervention; 111 spots vs. post-intervention; 116 spots).

## 4. Discussion

This study examined immune cell characteristics before and after an eight-week exercise intervention among breast cancer survivors, randomly allocated to a partly-supervised exercise group or a remotely-supported exercise group. Using multi-parameter flow cytometry, we assessed absolute counts of lymphocyte subtypes and basal unstimulated activation levels of T cells in peripheral blood. In addition, using Enzyme-Linked ImmunoSpot (ELISpot) assays, we examined T cell IFN-γ production in response to stimulation with virus and tumour-associated antigens. In summary, we showed that, although total leukocyte counts did not change with eight-weeks of exercise training, changes were evident among some leukocyte subtypes. CD4+ EMRA T cells significantly decreased in number and their activation levels declined across all participants, irrespective of intervention group. In addition, the partly-supervised group exhibited a return of the CD4+/CD8+ ratio towards more normal values and a significant increase in the numbers of CD16– Regulatory NK cells. Eight-weeks of exercise training had no effect on T cell IFN-γ production in response to stimulation with virus antigens from CMV and VZV or tumour-associated antigens including Mammaglobin, Survivin and Mucin-1.

The significant decrease of CD4+ EMRA T cells in blood following exercise training could be interpreted as a sustained redistribution of cells to tissues, as has been demonstrated previously (56, 57). Indeed, it has been hypothesized that repeated bouts of exercise could increase the numbers of effector T cells in tissues, which might facilitate cancer immunosurveillance, even in the absence of a clinically detectable tumour (30). In support, studies across a range of pre-clinical tumour models in mice have shown that compared to non-exercising mice, exercise bouts following tumour challenge lead to a greater tumour infiltration of several immune cell subtypes—including T cells—and smaller/slower tumour growth (58). Mechanistically, this finding could be explained by acute bouts of exercise mobilising tissue-homing cells into blood, which subsequently migrate to tissues in the hours after exercise, most likely being attracted to sites of inflammation, such as tumours. However, a finding which is harder to explain, is that in the same study (58), mice undertaking exercise bouts for 4 weeks prior to tumour challenge (but not after) exhibited a greater tumour-infiltration of immune cells and smaller/slower tumour growth, than non-exercising mice. A possible explanation is a gradual exercise-induced accumulation of effector cells in tissues, so that these cells are ready to respond to malignant transformation if encountered. Indeed, the delivery and accumulation of these immune cells within tissues could be influenced by an exercise-induced increase in pan-tissue vascularization (59, 60). Thus, in the present study, the significant decrease of CD4+ EMRA T cells in blood—which exhibit strong tissue-homing potential—could indicate, in humans, that regular exercise increases the trafficking of immune cells from blood to tissues, as part of immune surveillance (61–64). If proven directly, then this could be one mechanism for how regular exercise reduces the risk of cancer overall, and prevents cancer recurrence, by facilitating the detection and elimination of pre-malignant or malignant cells in tissues (30).

An alternative interpretation for the fall in CD4+ EMRA T cells in blood after eight weeks of exercise training is an exercise-induced anti-immunosenescence effect, which is thought to be driven by three processes. First, cells of a late-stage differentiated phenotype are mobilised into blood during exercise bouts. Second, these cells extravasate from blood, homing to tissues, where they are exposed to pro-apoptotic stimuli. Third, the naïve T cell pool expands, due to exercise-induced thymopoiesis and/or extrathymic T cell development in tissues such as the liver (65–67). Several studies have provided evidence in support of this hypothesis. For example, a study in 32 healthy young women aged 18–29 years examined exercise-induced changes in T, B and NK cell subtypes in blood (68). Participants undertook either a weekly 90 min session of aerobic-type total-body-shaping workouts (TBSW) or Pilates workouts (PW) for 14 weeks. The TBSW group showed increases in the percentage of naïve B cells and CD8+ NA T cells (*p* < 0.0428), while CD4+ CM T cells and CD8+ EMRA T cells decreased (*p* < 0.0363) (68). Evidence for an anti-immunosenescence effect of exercise training has also been shown in older populations. For example, 100 healthy older (>65 years) women were randomly assigned to strength endurance training (SET, 40% one-repetition maximum), intensive strength training (IST, 80% one-repetition maximum) or a control group (CON, stretching exercises) (69). The SET group exhibited a significant decline in the numbers and proportions of so-called “senescent prone” CD8+ T cells, defined using CD57 and CD28 (*p* < 0.05) (69). In addition, 29 older (>65 years) sedentary women who took part in a 6 week-long functional conditioning gymnastic exercise program exhibited a significant increase in the proportions of CD8+ NA and CM cells (*p* < 0.0408) and a significant decrease of CD8+ EMRA T cells (*p* = 0.0238) (70). Further support is shown by a study that randomized 40 inactive people aged 60–75 years into either a 6-week low-dose combined resistance and endurance training exercise group or a control group (71). Exercise training returned the CD4+/CD8+ ratio to normal values of between 1.5 and 2.5 (*p* = 0.043), whereas the control group exhibited increases in “senescent-prone” CD8+ T cells defined using CD57 and CD28 (*p* < 0.006) (71). Evidence in clinical populations is lacking, other than a small study of 16 men diagnosed as having pre-diabetes (72). In this study, men were randomised to a 3-week concentric exercise (CE) or eccentric exercise (EE) resistance training group. It was shown that both groups exhibited a significant increase in the proportions of CD8+ NA, CD4+ CM and CD8+ CM (*p* < 0.047) and a significant fall in the proportions of CD4+ and CD8+ EMRA T cells (*p* < 0.018) (72). Thus, the present study is novel, by providing evidence of a possible anti-immunosenescence effect of exercise among cancer survivors.

In addition to being a hallmark of an ageing immune system, high numbers of late differentiated T cells have been associated with poor clinical outcomes in cancer settings. For example, in a study recruiting 89 women with metastatic breast cancer, it was shown that higher frequencies of CD8+ CD28− cells was negatively correlated with progression free survival (73). Patients with ≥24.0% of these cells within the CD8+ T cell pool exhibited median survival of 2 months less compared to patients with a lower frequency (<24.0%) (*p* < 0.001) (73). Thus, whether the fall in CD4+ EMRA T cells in the present study is interpreted as either cell trafficking and accumulation in tissues, perhaps facilitating cancer immune-surveillance, *or* whether this finding is interpreted as an anti-immunosenescence effect of exercise, *both* interpretations could have clinical relevance. In other words, if exercise decreases the counts of late-stage differentiated T cells—which could be defined using many overlapping strategies (74, 75)—then this effect could lead to improvements in recurrence and survival. Although the concept of exercise-induced apoptosis of late-stage differentiated T cells in tissues might at first seem to be in stark contrast to an accumulation of tissue-resident effector cells searching for pre-malignant cells, there is an important nuance. It has been proposed that the late-stage differentiated effector cells selectively removed by exercise are not needed. For example, at least some (e.g., up to 10%) of the T cells with a CD4+ EMRA phenotype in the present study will have been specific for the latent herpes virus *Cytomegalovirus* (CMV) (76). If regular exercise helps to limit viral reactivation by promoting redox balance and an anti-inflammatory environment (77), then it could be speculated that fewer CMV-specific T cells are needed. In support, we show a statistically significant fall in CMV-specific IgG after exercise training, which is considered to be a proxy marker of viral reactivation (78, 79). Similarly and in support, a 6-weeks endurance exercise training study in older (>65 years) women found a significant decrease in the number of “senescence-prone” T cells, but also showed a significant positive correlation between the number and the proportion of these cells and the concentration of CMV IgG (80).

Further evidence from this study supports an anti-immunosenescence effect of exercise. We show a statistically significant group × time interaction effect for a change in the CD4+/CD8+ ratio (*p* = 0.021). This effect was driven by a statistically significant fall in the CD4+/CD8+ ratio among members of the partly-supervised exercise group (*p* = 0.006). Indeed, from this group, a third of breast cancer survivors with a high ratio (>2.5) exhibited a return to a normal ratio, of between 1 and 2.5. This is a promising finding, given that an inverted (<1) or high (>2.5) CD4+/CD8+ ratio has been associated with immunosenescence, the immune risk profile (20) and chronic inflammation (51–53). Further, a very high CD4+/CD8+ ratio has been linked with frailty and poor survival (81). Similar findings have been shown in non-cancer settings. For example, a cross sectional study among adults who were 60–90 years of age, classified participants as being physically active or physically inactive using a combination of directly measured cardiorespiratory fitness and lifestyle questionnaires (53). It was shown that there was a lower percentage of people defined as being physically active with inverted (<1) or increased (>2.5) CD4+/CD8+ ratios compared to people being defined as physically inactive group (active: 44% vs. inactive: 55%).

A strength of this investigation is that we examined a range of lymphocyte populations in blood that have received interest in ageing, cancer and exercise settings. For example, stem cell like memory T cells (TSCMs)—which are thought to have anti-tumour potential (82)—were examined, but showed no differences with eight weeks of exercise training. In addition, total B cells, memory B cells, immature B cells and naïve B cells and plasmablasts were also examined, and exhibited small non-statistically significant fluctuations in number following training, with divergent trends between groups (i.e., the partly-supervised group exhibited increases, whereas the remotely-supported group exhibited decreases). B cells have been shown to have roles in anti-tumour immunity, by facilitating the optimal stimulation and clonal expansion of T cells (83, 84). In addition, low B cell numbers are part of the immune risk profile (20) and B cell number and function generally decline with immunosenescence (85). Finally, we also showed that the numbers of NK cells and their subtypes did not substantially change with eight weeks of exercise training, although there was a trend for these cells to increase at a whole group level and in the partly-supervised group. Furthermore, CD16− Regulatory NK cells were significantly increased in the partly-supervised group following training. Previous studies have shown that NK cell numbers and cytotoxicity are increased after moderate-intensity continuous training and high-intensity interval training among untrained healthy people (86). Indeed, in the context of the present study, higher NK cell counts have been associated with good prognosis in several cancers (87–89). However, a recent systematic review of studies examining the effects of exercise training on the numbers and functions of NK cells among breast cancer survivors could not find conclusive evidence of a consistent and beneficial effect (90).

T cell activation is essential for a targeted immune response (91) and is positively associated with survival in cancer settings (92, 93) and a good response to treatment (94). Our assessments of basal non-stimulated T cell activation using HLA-DR expression generally showed no change with eight weeks of exercise training. However, for CD4+ EMRA T cells, there was a statistically significant decrease in HLA-DR expression density with training, which could be interpreted as an anti-inflammatory response to exercise. Indeed, chronic “non-specific” T cell activation has been linked to inflammatory processes that can contribute to the development of malignancy and cancer recurrence (95). An alternative explanation for the lower HLA-DR expression on CD4+ EMRA T cells in blood following eight weeks of exercise training is the gradual accumulation of cells in tissues, so that the most activated cells are no longer present in blood (61–64).

For the first time, the present study examined whether T cell IFN-γ production in response to stimulation with virus and tumour-associated antigens changed with eight weeks of exercise training among breast cancer survivors. Although there were no statistically significant changes in antigen-specific T cell responses with training, there was an overall trend for an increase in the response to VZV, Mammaglobin, Mucin-1 and Survivin, especially in the partly-supervised exercise group. T cell IFN-γ production in response to stimulation with CMV pp65 was lower following training, aligning with the idea of better anti-viral control with regular exercise, and supporting our finding of lower CMV-specific IgG following training (77, 80). Strong T cell responses to tumour associated antigens measured in blood is positively associated with good clinical outcomes. For example, in a study of 40 patients with breast cancer, those who had HER2-reactive T cells and a lower proportion of Myeloid derived suppressor cells (MDSCs) before treatment exhibited a 100% rate of survival after 5 years, compared to 38% survival among patients without HER2-reactive T cells and higher frequencies of MDSCs (*p* = 0.03). Similarly, 100% survival over 5 years was exhibited by patients with HER2-reactive T cells and lower frequencies of regulatory T cells compared to 50% survival among patients without HER2-reactive T cells and with higher levels of regulatory T cells (*p* = 0.03) (41). Thus, if regular exercise training can increase the numbers of tumour-reactive T cells in blood, or their magnitude of response, this could be one mechanism by which exercise reduces cancer recurrence and mortality.

The present study did not feature a non-exercise control group. Randomised and controlled trials comparing an exercise group to a non-exercise control group have shown that similar immunological measurements to those examined in the present study, are susceptible to exercise-induced change (e.g. 69, 71). Other studies have shown that exercise training among breast cancer survivors, compared to non-exercise controls, can positively modulate other aspects of immunity, including neutrophil function (e.g. 96). The present study examined whether the magnitude of exercise-induced immunological change (compared to pre-intervention values) was greater with a rigorously controlled intervention (i.e., the partly-supervised group) compared to an intervention that would be more feasible for implementation in clinical settings (i.e., the remotely-supported group). Inclusion of a non-exercise control group would have enabled analyses to isolate the effects of exercise *per se* and would eliminate possible confounding by time (e.g., week-by-week variation). However, changes in immune cell characteristics over eight weeks in the absence of a change in lifestyle or disease are likely to be very small in magnitude (97). It should also be considered that cryopreserved cells were examined in the present study, which may have impacted the phenotypic or functional characteristics of cells present in samples. Although cell viability was >90% and results from positive and negative controls were interpreted carefully, the magnitude of IFN-γ production in functional assays is likely to have been smaller than with fresh samples, however it is unlikely that the pattern of change and overall study results would have been affected. Finally, given the small sample size, the results of the present study provide proof-of-principle evidence that immune cell characteristics and function are susceptible to exercise-induced change among breast cancer survivors.

In summary, we showed that eight-weeks of exercise training decreased the counts and activation levels of CD4+ EMRA T cells among breast cancer survivors. In addition, the partly-supervised exercise group exhibited a return of the CD4+/CD8+ ratio towards more normal values and an increase in the numbers of CD16− Regulatory NK cells. Eight-weeks of exercise training had no effect on T cell IFN-γ production in response to stimulation with virus antigens from CMV and VZV or tumour-associated antigens including Mammaglobin, Survivin and Mucin-1. However, the response to tumour-associated antigens was maintained or at least slightly larger in magnitude following exercise training. Our results add to the large body of literature, suggesting that exercise is beneficial for breast cancer survivors. Our data could be interpreted as providing evidence of an anti-immunosenescence effect of exercise. Indeed, these changes might elicit changes in the immunological landscape that could have positive implications for anti-cancer immunity, giving further impetus for examining whether these exercise-induced changes can be linked to clinical outcomes in cancer settings.

## Data Availability

The raw data supporting the conclusions of this article will be made available by the authors, without undue reservation.
