# Supplementary material for: The effects of exercise training for eight weeks on immune cell characteristics among breast cancer survivors
Source: Front Sports Act Living. 2023 May 11;5:1163182. doi: 10.3389/fspor.2023.1163182 (PMC10211347; doi:10.3389/fspor.2023.1163182)
Supplement: Supplementary file 1 [file Table1.docx]

**Supplementary Table 1. Diagnostic and treatment information (extended).**

|  | **Diagnosis** | **Months since diagnosis** | **Months since surgery** | **Treatment** | | | |
| --- | --- | --- | --- | --- | --- | --- | --- |
|  |  |  |  | **Surgery** | **Chemotherapy** | **Radiotherapy** | **Endocrine therapy** |
| 1 | G3 T2 N1 ER/PR+ HER2+ | 19 | 17 | Lumpectomy | FEC-TH (adj) | yes | Type 1 |
| 2 | G3 T1 N0 multifocal ER/PR- HER2+ | 11 | 8 | Mastectomy | FEC-TH | - | - |
| 3 | G2 T1 N0 ductal ER/PR+ HER2- | 19 | 15 | Lumpectomy | - | yes | Type 1 |
| 4 | DCIS (Tis N0) ER/PR+ HER2- | 15 | 14 | Lumpectomy | - | yes | - |
| 5 | G2 T2 N0 ER/PR+ HER2- | 7 | 6 | Lumpectomy | - | yes | Type 3 |
| 6 | G2 T1 N0 multifocal (with DCIS) ER/PR+HER2- | 13 | 11 | Mastectomy | FEC-T | - | Type 2 |
| 7 | G2 T2 N0 ER/PR+ HER2- | 10 | 8 | Lumpectomy | - | yes | Type 2 |
| 8 | G3 T1 N1 ER/PR+ HER2- | 8 | 6 | Lumpectomy | - | yes | Type 1 |
| 9 | G3 T2 N1 ER/PR- HER2- | 12 | 11 | Mastectomy | FEC-T | yes | - |
| 10 | DCIS (Tis N0) | 9 | 8 | Lumpectomy | - | yes | - |
| 11 | DCIS (Tis N0) * | 20 | 19 | Mastectomy | - | - | - |
| 12 | DCIS (Tis N0) | 22 | 21 | Lumpectomy | - | yes | - |
| 13 | G2 T1 N0 ER/PR+ HER2- | 26 | 25 | Lumpectomy | - | yes | Type 3 |
| 14 | DCIS (Tis N0) ER/PR+ HER2- | 14 | 9 | Mastectomy | - | - | Type 1 |
| 15 | G2 T3 N3 ER/PR+ HER2- | 29 | 23 | Mastectomy | FEC-T (neoadj) | yes | Type 3 |
| 16 | G2 T1 N2 (with DCIS) ER/PR+ HER2- | 9 | 6 | Mastectomy | - | yes | Type 1b |
| 17 | G2 T2 N0 ER/PR+ HER2- | 7 | 5 | Lumpectomy | - | yes | Type 1 |
| 18 | G3 T2 N0 ER/PR- HER2- | 7 | 7 | Lumpectomy | - | yes | - |
| 19 | DCIS (Tis N0) | 19 | 16 | Mastectomy | - | - | - |
| 20 | G2 T1 N0 ER/PR+ HER2- | 7 | 5 | Lumpectomy | - | yes | Type 1 |

***Legend for Supplementary Table 1:*** *G (grade) refers to the Histologic grade (G1, G2, G3, etc). T (tumour) and N (nodes) values refer to the TNM staging to define the characteristics of the tumour (T) 0 to 4 (is=in situ) and node (N) involvement 0 to 3.* ***Other abbreviations key:*** *adj=adjuvant, DCIS= Ductal Carcinoma In Situ, neoadj= neoadjuvant.* ***Treatment key:*** *FEC-T= Fluorouracil, Epirubicin and Cyclophosphamide for 3 cycles followed by Docetaxel for 3 cycles; FEC-TH= Fluorouracil, Epirubicin and Cyclophosphamide for 3 cycles followed by Docetaxel for 3 cycles, plus Herceptin.* *Type 1= Endocrine therapy regimen 1: letrozole (aromatase inhibitor), Type 1b= Endocrine therapy regimen 1b: letrozole (aromatase inhibitor) plus LHRHa, Type 2= Endocrine therapy regimen 2: anastrozole (aromatase inhibitor), Type 3= Endocrine therapy regimen 3: tamoxifen. *= This patient had a prior breast cancer diagnosis in 1995 but was treated elsewhere; no more detail is known about this previous diagnosis and/or treatment.*
